# Short-term health-related quality of life consequences in a lung cancer CT screening trial (NELSON)

**DOI:** 10.1038/sj.bjc.6605459

**Published:** 2009-11-24

**Authors:** K A M van den Bergh, M L Essink-Bot, G J J M Borsboom, E Th Scholten, M Prokop, H J de Koning, R J van Klaveren

**Affiliations:** 1 Department of Public Health, Erasmus MC, University Medical Centre Rotterdam, Rotterdam, The Netherlands; 2 Department of Social Medicine, Academic Medical Centre, University of Amsterdam, Amsterdam, The Netherlands; 3 Department of Radiology, Kennemer Gasthuis Haarlem, Haarlem, The Netherlands; 4 Department of Radiology, University Medical Center Utrecht, Utrecht, The Netherlands; 5 Department of Pulmonology, Erasmus MC, University Medical Centre Rotterdam, Rotterdam, The Netherlands

**Keywords:** lung neoplasms, mass screening, quality of life, spiral computed tomography

## Abstract

**Background:**

In lung cancer CT screening, participants often have an indeterminate screening result at baseline requiring a follow-up CT. In subjects with either an indeterminate or a negative result after screening, we investigated whether health-related quality of life (HRQoL) changed over time and differed between groups in the short term.

**Methods:**

A total of 733 participants in the NELSON trial received four questionnaires: T0, before randomisation; T1, 1 week before the baseline screening; T2, 1 day after the screening; and T3, 2 months after the screening results but before the 3-month follow-up CT. HRQoL was measured as generic HRQoL (the 12-item Short Form, SF-12; the EuroQol questionnaire, EQ-5D), anxiety (the Spielberger State-Trait Anxiety Inventory, STAI-6), and lung-cancer-specific distress (the Impact of Event Scale, IES). For analyses, repeated-measures analysis of variance was used, adjusted for covariates.

**Results:**

Response to each questionnaire was 88% or higher. Scores on SF-12, EQ-5D, and STAI-6 showed no clinically relevant changes over time. At T3, IES scores that were clinically relevant increased after an indeterminate result, whereas these scores showed a significant decrease after a negative result. At T3, differences in IES scores between the two baseline result groups were both significant and clinically relevant (*P*<0.01).

**Conclusion:**

This longitudinal study among participants of a lung cancer screening programme showed that in the short term recipients of an indeterminate result experienced increased lung-cancer-specific distress, whereas the HRQoL changes after a negative baseline screening result may be interpreted as a relief.

Lung cancer is the main cause of cancer-related deaths worldwide among men and women (Ferlay *et al*, 2004; American Cancer Society, 2007). Although cancer can be detected in an early stage by computed tomography (CT) screening (Henschke *et al*, 2006), results from randomised controlled trials are needed before deciding whether CT screening will reduce lung cancer mortality, and whether implementation of large-scale lung cancer CT screenings programmes should be recommended (Gohagan *et al*, 2004; Bach *et al*, 2007; van Iersel *et al*, 2007; Field and Duffy, 2008).

Most CT screening studies report baseline rates of 14–43% of non-calcified nodules (5–10 mm in diameter); this relatively large range is attributed to geographic differences in nodule prevalence and the slice thickness used (Diederich *et al*, 2002; Wilson *et al*, 2008). Subjects with this type of nodule usually receive a recommendation to undergo a follow-up CT 3–4 months later to assess whether a nodule has grown, because nodule growth is associated with increased cancer risk (Swensen *et al*, 2005).

In the Dutch–Belgian randomised controlled trial for lung cancer screening in high-risk subjects (the NELSON trial), subjects could receive either a negative, an indeterminate, or a positive scan result (Xu *et al*, 2006). Subjects receiving an indeterminate scan result at baseline were invited to undergo a follow-up scan 3 months later; however, receiving such a result and waiting for this scan might have an unfavourable effect on health-related quality of life (HRQoL), compared with receiving a negative result. For example, in the PLuSS study, a significant increase of generic anxiety was found 1–2 weeks after communication of an indeterminate baseline screening result (Byrne *et al*, 2008). However, Byrne *et al* (2008) used HRQoL instruments that precluded detailed evaluation of the psychological consequences of lung cancer screening; moreover, possible changes in HRQoL between the baseline test result and the 3-month follow-up scan result were not reported. Furthermore, a study on breast cancer screening showed that anxiety was higher just before screening, compared to basic HRQoL unrelated to screening (Rijnsburger *et al*, 2004). So, to determine the whole effect of screening, it is important to establish whether HRQoL is already negatively affected just before baseline screening.

In this study we assessed changes in generic and lung-cancer-specific HRQoL changes over time among participants undergoing lung cancer screening in the short term. Therefore, we addressed the following questions: (1) To what extent does HRQoL decrease just before baseline screening? (2) Is there a difference in HRQoL between those with an indeterminate baseline result and those with a negative result? We hypothesised that lung-cancer-specific distress scores just before baseline CT screening would be higher compared with scores acquired a few months before screening (Rijnsburger *et al*, 2004). Also, in subjects who received an indeterminate baseline result we expected higher levels of lung-cancer-specific distress 2 months after screening (but before the 3-month follow-up scan) compared to those who received a negative result.

## Materials and methods

### NELSON study population

A random sample of Dutch and Belgian subjects (aged 50–75 years) registered in population registries received a questionnaire containing items about health and smoking history. Current and former smokers were asked to complete this ‘first’ NELSON questionnaire. Respondents who had smoked >15 cigarettes per day for >25 years or >10 cigarettes per day for >30 years, those who still smoked, or those who had quit 10 or less years ago were invited to participate in the trial (van Iersel *et al*, 2007).

Informed consent was obtained from 15 822 high-risk subjects who were subsequently randomised (1 : 1) to either a screening group, or a control group that received no screening. Participants in the screening group could receive either a positive, indeterminate, or negative test result within 3 weeks after the baseline CT scan was performed (Xu *et al*, 2006). A positive test result required referral to a pulmonologist for work up and diagnosis.

Participants with an indeterminate result were scheduled to undergo a follow-up CT scan 3 months later to evaluate whether the nodule had grown. The letter to participants with an indeterminate result stated: ‘… we have observed a very small abnormality in your lung (5 to 10 mm long). Such a small abnormality is often detected in many persons and it usually represents a small scar or a minor inflammation. Therefore, at this moment there is no need for any further investigations. However, in order to see whether there has been any change in this abnormality, a new CT scan of the lungs will be made after 3 to 4 months.’ The letter also explains the possible results and related work-up after this follow-up CT scan: ‘… participants with an abnormality showing no growth will receive a negative test result and will be invited for a CT scan 1 year after the baseline screening. Those with an abnormality showing some growth will be referred to a pulmonologist for further investigations’ (Xu *et al*, 2006).

The NELSON trial, including the current HRQoL study, was approved by the Dutch Ministry of Health and by the local ethics committees of the participating centres. Informed consent was obtained from all participants. The NELSON trial is registered at www.trialregister.nl with number ISRCTN63545820.

### HRQoL study

A consecutive sample of 1466 participants was taken from the screening centres in Haarlem and Utrecht, randomised in August 2005 (*n*=977), September 2005 (*n*=390), and November 2005 (*n*=99). All participants received a questionnaire after eligibility check, after sending the information brochure, and signing of the informed consent form, but before trial randomisation (Time 0 (T0), baseline HRQoL assessment). Subjects randomised to the screen arm received a second questionnaire 1 week before the baseline scan (Time 1, T1); they were asked to complete the questionnaire before the baseline scan was performed. At 1 day after this baseline scan, they received a third questionnaire (Time 2, T2) and were asked to complete this questionnaire within 1 week. At T2, subjects did not receive the scan result of the baseline scan. Finally, for subjects who had a negative or an indeterminate test result, a questionnaire was sent about 2 months after the baseline scan was made (Time 3, T3). For subjects with an indeterminate scan result this was about 1 month before the 3-month follow-up scan.

In this study, the response of those who did not undergo baseline screening, or who had a positive test result, was excluded from the analyses. The questionnaire responses of those who completed T1 after the CT scan (*n*=12), who completed T2 before the CT scan (*n*=0) or after the baseline test result (*n*=6), and who completed T3 after the result of the follow-up scan (*n*=1) were excluded. These were not counted as responses.

### Measures

#### Generic HRQoL

The participant's generic HRQoL was measured with the 12-item Short Form (SF-12) and the EuroQol questionnaire (EQ-5D) (Essink-Bot *et al*, 1993; Ware *et al*, 1996; Gandek *et al*, 1998; Kind *et al*, 2005). The SF-12 is a shorter version of the SF-36 and consists of a physical component summary (PCS) and a mental component summary (MCS) (Ware *et al*, 1996). We used the acute (1-week recall) form of version 1. Each participant completed the SF-12 at T0 and T3. A higher score indicates a better HRQoL.

Respondents were also asked to rate their own health on the visual analogue scale (VAS) of the EQ-5D, ranging from 0 (worst imaginable health status) to 100 (best imaginable health status) (Essink-Bot *et al*, 1993; Kind *et al*, 2005). Participants completed the EQ-5D VAS at all four assessment points (i.e. T0, T1, T2, and T3).

#### Generic anxiety

Generic anxiety was measured using the short form of the Spielberger State-Trait Anxiety Inventory (STAI-6) (van der Bij *et al*, 2003). Six items related to anxiety (calm, tense, upset, relaxed, content, and worried) were rated on a four-point scale. The total summary score was calculated in subjects with a maximum of three missing values and could range from 20 to 80, with higher scores indicating more anxiety (Maissi *et al*, 2005). The STAI-6 is reported to have good reliability and validity, and was found useful to evaluate the effectiveness of screening programmes on subjective anxiety levels (van der Bij *et al*, 2003). The STAI-6 was used at all four assessment points.

#### Lung-cancer-specific distress

Lung-cancer-specific distress was measured using the Impact of Event Scale (IES) (Horowitz *et al*, 1979; Brom and Kleber, 1985). The 15 IES items were tailored to lung cancer as the specific stressors. Each item was scored on a four-point scale: not at all (score of 0), rarely (score of 1), sometimes (score of 3), and often (score of 5). The total score and subscales (avoidance and intrusion) were calculated for those who completed 75% of the questions on each subscale, and were corrected for the total number of questions on the subscale. The total summary score could range from 0 to 75 (intrusive scale 0–35, avoidance scale 0–40), with a higher score indicating more lung-cancer-specific distress. The IES was used at all four assessment points.

#### Demographic and other data

At T0, the questionnaire had items on marital and smoking status. Educational level and smoking pack-years were derived from the first NELSON questionnaire.

### Statistical analyses

Differences in respondent characteristics between those with a negative or indeterminate baseline scan result were tested with Mann–Whitney *U*-tests (in case of non-normally distributed continuous variables) and *χ*^2^-tests (for discrete variables). Then, we first analysed differences in HRQol over time, focusing on differences between the two baseline result groups. Second, the changes in HRQoL before and after the baseline scan result were analysed. For the latter analyses, we started by using data of the total group in the period before the CT scan result (T0, T1, and T2), because all subjects were still unaware of the baseline CT result. After the CT scan result (T0–T3 and T2 and T3) the data from the two result groups were analysed separately. For all analyses repeated-measures analysis of variance (ANOVA) was applied, using ‘proc mixed’ from the SAS system version 9.1 (SAS Institute Inc., Cary, NC, USA); this allowed use of all available data, including the incomplete records. For the subjects, we used models with a random intercept to allow for dependence between the repeated measurements.

#### Effect of baseline result on HRQoL over time

Differences in HRQoL between the negative and indeterminate result groups were analysed at the four assessment points. The models included a main effect for time, and for an interaction between group and time. Time was included as a factor with four levels (one for each assessment) to account for possible non-linearity in the change in HRQoL scores. The following fixed covariates were added to the model: age (because older people are reported to show less anxiety and better mental health) (Taylor *et al*, 2004; Byrne *et al*, 2008), gender (because women are reported to show a different fear of cancer and have worse generic HRQoL compared with men) (Taylor *et al*, 2004), education (because higher-educated lung cancer screening participants are reported to be less anxious, have less fear of cancer and less distress) (Byrne *et al*, 2008), smoking status (because current smokers generally have a worse HRQoL than non-smokers, and more anxiety and fear of cancer) (Laaksonen *et al*, 2006), and smoking pack-years (because we expected subjects with more pack-years to be more anxious and to have worse health).

The IES scores were highly skewed. However, as a logistic regression model using a generalised linear mixed models approach analysis would limit the data, and because choosing a cut-off point is arbitrary and use of the model in fact produced the same results as with the repeated-measures ANOVA, we considered repeated-measures ANOVA to be appropriate for the IES scores.

#### Change in HRQoL before and after receipt of baseline scan result

Before the receipt of the baseline result (T0, T1, and T2), the same repeated-measures models as described above (adjusting for covariates) were used for the total group, but this time including contrasts to test differences in scores of the total group between specific assessment points (ie T0 *vs* T1, and T1 *vs* T2, and a model with T0 *vs* T2). In these models, the main effect for group, and the interaction between group and time, was no longer necessary and was thus excluded. After receipt of the baseline result (T3) changes in HRQoL between T2 and T3 and between T0 and T3 were analysed separately for the groups with a negative and with an indeterminate baseline result. The same repeated-measures model was used as for the analyses between T0, T1, and T2.

A *P*-value <0.05 was considered statistically significant. To provide a clue to the meaningfulness of statistically significant differences between means at two assessments or between subgroups, we used the minimal important difference (MID), which is defined as half of a standard deviation (s.d.) of the mean (Norman *et al*, 2003). The MID can serve as a default value for meaningful changes in HRQoL. For changes over time the s.d. at the first assessment of the two compared assessment points was used, and for differences between groups the pooled s.d. of the two groups at a specific time point was used.

## Results

### Response and respondent characteristics

**Figure 1:**
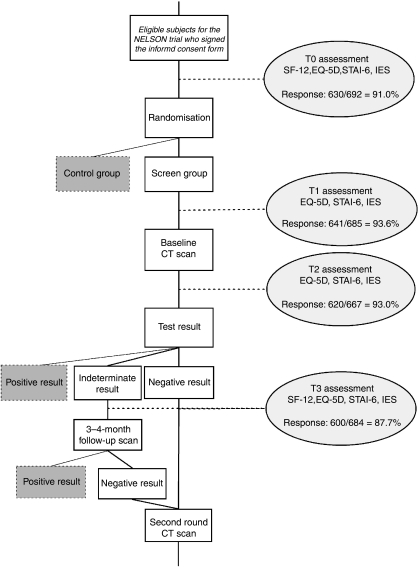
Flow chart of the HRQoL study. 41 subjects out of 733 of the screen group were excluded from the HRQoL study: 30 had no baseline CT scan, and 11 had a positive CT result at baseline. At T1, T2, and T3 a total of 7, 25, and 8 questionnaires, respectively, were not sent due to administrative failures. Responses at T1, T2, and T3 were excluded for 1, 2, and 2 questionnaires, respectively, due to more than 50% missing items. Also excluded were T1 questionnaires (*n*=10) completed after the baseline CT scan, T2 questionnaires (*n*=6) completed after the baseline CT scan result, and T3 questionnaires (*n*=1) completed after the follow-up scan result.

In total, 41 screen arm participants (5.6%) were excluded from the HRQoL study because they either did not undergo baseline screening (*n*=30) or had a positive baseline result (*n*=11). In the screen group, the response to the questionnaires was 91.0% (630 out of 692) at T0, 93.6% (641 out of 685) at T1, 93.0% (620 out of 667) at T2, and 87.7% (600 out of 684) at T3 (Figure 1). At least one of the four questionnaires was returned by 99.6% (689 out of 692) of the subjects, and 71.4% (494 out of 692) completed all four questionnaires. The T0 questionnaire was completed 164.8 (s.d. 107.5) days before baseline screening, and the T1 questionnaire 2.5 (s.d. 6.5) days before baseline screening. The T2 questionnaire was completed 4.0 (s.d. 3.3) days after baseline screening, and the T3 questionnaire 80.2 (s.d. 20.1) days after baseline screening. The T3 questionnaire was completed 59.4 (s.d. 24.1) days after the baseline screening result. For subjects with an indeterminate result this was 20.1 (s.d. 16.3) days before the follow-up scan.

Almost 50% of the respondents were men and the mean age was about 58 years (Table 1). No statistically significant differences in background characteristics were found between subjects with a negative and indeterminate baseline screening results.

### Effect of baseline result on HRQoL over time

**Figure 2:**
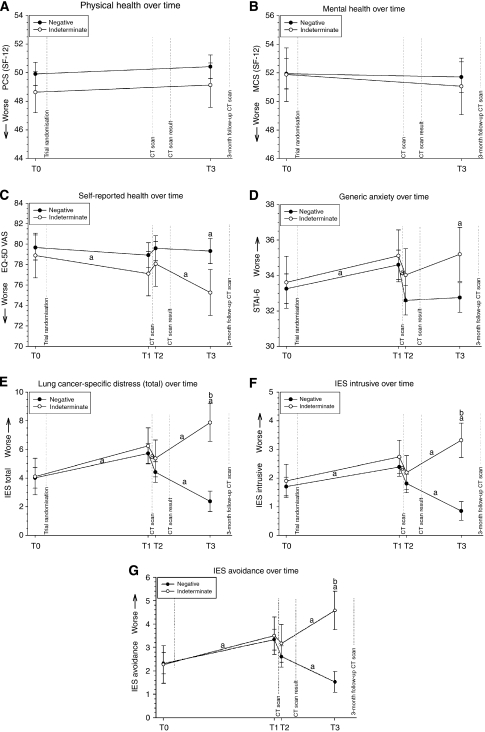
(**A–G**) Average scale scores and 95% confidence intervals per result group (negative or indeterminate baseline result) adjusted for gender, age, education, smoking status, and smoking pack-years: SF-12 (PCS and MCS) (**A** and **B**); EQ-5D VAS (**C**), STAI-6 (**D**), and IES (total, intrusive, avoidance) (**E**–**G**) T0, before trial randomisation; T1, just before baseline CT scan; T2, 1 day after baseline CT scan; and T3, about 2 months after baseline CT scan. ^a^Significant difference. ^b^Clinically relevant difference. (**A**–**C**) A higher score indicates better HRQoL. (**D**–**G**) A lower score indicates better HRQoL.

At each assessment, subjects with a negative test result had better HRQoL scores on all scales than subjects with an indeterminate result (Table 2; Figures 2A–G). Results of the repeated-measures analysis (adjusted for gender, age, education, smoking status, and smoking pack-years) showed no statistically significant differences in the SF-12 scores (MCS and PCS) between subjects with a negative and an indeterminate result at the two assessment points (Figures 2A and B). Also, at T0, T1, and T2 no statistically significant differences were found in EQ-5D-VAS and STAI-6 scores between subjects with a negative and an indeterminate test result (Figures 2C and D). At T3, compared with subjects with a negative result, those with an indeterminate result had statistically significantly lower scores on the EQ-5D-VAS and higher scores on the STAI-6 (i.e. both worse), but the difference was not clinically relevant (both *P*'s <0.01). At T0, T1, and T2 the IES total score showed no statistically significant inter-group difference, whereas at T3 the IES scores in the indeterminate result group were statistically significant and clinically relevant higher (i.e. worse) than in the negative result group (*P*<0.01) (Figures 2E–G).

In women and current smokers, scores on the PCS, EQ-5D VAS, STAI-6, and IES showed a statistically significant difference, but were not clinically relevant worse (i.e. they did not exceed the MID), compared with men and former smokers (data not shown).

### Change in HRQoL before and after receipt of baseline scan result

Before the receipt of the baseline scan result, HRQoL scores on the EQ-5D VAS, STAI-6, and IES for the total group of respondents were statistically significantly worse at T1 (just before the baseline CT scan) compared with those at T0 (*P*<0.05 for EQ-5D, rest < 0.01) (Figures 2A–G). Between T1 and T2, there was no statistically significant change in EQ-5D VAS scores. Average scores on the STAI-6 and IES were statistically significantly better at T2 (just after the CT scan) compared with T1 (all *P*<0.01). IES total scores and the IES avoidance scores did not revert to baseline levels, as they were statistically significantly worse at T2 compared with T0 (both *P*'s <0.05). In the total group, none of the statistically significant changes over time exceeded the MID, thus none of them was clinically relevant.

In the negative result group, the EQ-5D VAS and STAI-6 scores remained unchanged between T2 and T3, and between T0 and T3. The IES scores were statistically significantly lower (ie better) at T3 compared with T2 (all *P*<0.01) and also compared with T0 (*P*<0.01). In the indeterminate result group, the EQ-5D and the IES scores were worse at T3 compared with T2 (*P*<0.01), and compared with T0 (*P*<0.01). The STAI-6 scores remained unchanged between T2 and T3, but were worse at T3 compared with T0 (*P*<0.05). For all statistically significant differences in HRQoL over time, only the changes in IES scores between T0 and T3 in the indeterminate result group were also clinically relevant.

### Impact of covariates on HRQoL

In general, the HRQoL scores were worse for women than for men (*P*<0.05). Subjects with more pack-years had a worse self-reported health (EQ-5D VAS) and had worse physical health scores (PCS) than subjects with less pack-years (*P*<0.05). Current smokers had worse HRQoL scores at all scales (*P*<0.05) except for the mental health scores (MCS) than former smokers.

## Discussion

Lung-cancer-specific distress increased in a clinically relevant manner 2 months after receipt of an indeterminate result of baseline screening. After receiving the baseline CT result, subjects with an indeterminate screening result had clinically relevant higher lung-cancer-specific distress than subjects with a negative result. In the groups with a negative or indeterminate result, no clinically relevant differences over time within or between groups were found for physical/mental/self-reported health and generic anxiety.

In this study, the statistically significantly worse HRQoL just before the CT scan, compared with HRQoL at a neutral point of time before screening (T0), is similar to an earlier report on breast cancer screening (Rijnsburger *et al*, 2004); however, in the latter study it is unknown whether the self-reported health change exceeded the MID of half an s.d. As a result of a slightly unfavourable effect of CT scanning on HRQoL, we did not find any clinically relevant changes between the assessment points (T0 to T1 to T2 to T3). Nevertheless, in the indeterminate result group there was a clinically relevant increase in lung-cancer-specific distress when comparing T0 with T3. This implies that performing an HRQoL assessment at a neutral point in time is important.

In our indeterminate result group, the STAI anxiety scores showed a statistically significant increase from the baseline HRQoL assessment up to 2 months after receipt of the baseline screening result. Byrne *et al* (2008) also found a statistically significant increase in anxiety 1–2 weeks after an indeterminate baseline result compared with before the CT scan. However, the size of the change was below our criterion for clinical relevance. Re-evaluation of the reported unadjusted means in the study of Byrne *et al* revealed that anxiety scores for indeterminates were not clinically relevantly worse, which is similar to our results. However, comparison of the results of the PLuSS study and ours was difficult because the details of their result letter to the participants were unknown, and the follow-up time for the indeterminate results also differed (Xu *et al*, 2006; Byrne *et al*, 2008; Wilson *et al*, 2008).

Using a more specific HRQoL instrument (i.e. the IES), we could show both a statistically significant and a clinically relevant change from the baseline HRQoL assessment up to 2 months after the receipt of the result, as well as a difference between our two result groups. This implies that an indeterminate test result had at least some negative impact on HRQoL in the period between receipt of the test result and the follow-up scan 3–4 months later. Nevertheless, the effect was small because the average IES total score in the indeterminate group was only 8.3 (s.d. 11.3) on a scale with an upper limit of 75. The IES scales were also highly skewed; even in the indeterminate result group 30% did not experience any lung-cancer-specific distress at 2 months after screening (i.e. IES total score=0).

The HRQoL decrement should be very low in a screening situation, because even a small HRQoL decrement due to screening at the individual level will accumulate to a large burden at population level due to the large numbers of subjects involved. By using the MID criterion, we intended to provide a clue to the meaningfulness of a statistically significant change in mean scores. An additional reason for using the MID was the fact that this study included large numbers of subjects and that HRQoL scale scores do not have an intuitive interpretation. It is situation dependent whether a statistically significant change in mean scores from, for example 12.1 to 11.7 is to be regarded as a meaningful difference.

Remarkably, in the indeterminate result group, at all assessment points the HRQoL scores were worse than those in the group with negative results. This was the case before the screening result was known, and even before screening took place; however, these differences were not statistically significant. Subjects who had a positive baseline CT scan who completed the T0 questionnaire (*n*=8) reported even worse HRQoL scores before screening (data not shown). Previous studies showed a prognostic effect of HRQoL on survival (of lung cancer) or disease onset (Montazeri *et al*, 2001). Our results suggest that worse HRQoL scores before screening may serve as a weak indicator of an indeterminate or positive baseline scan result.

In the indeterminate group, we did not assess HRQoL after they had received the result of the 3-month follow-up scan. This would have provided additional information on the further evolution of the unfavourable HRQoL scores in test indeterminates, especially because the majority would have received a negative result based on this follow-up scan. However, in our previous study on HRQoL we found no differences between subjects with a negative baseline CT scan and subjects with an indeterminate follow-up scan that had a negative follow-up CT scan (van den Bergh *et al*, 2008). It would have been interesting to evaluate the HRQoL effects in subjects who received a positive test result; however, because only 11 subjects received a positive result at baseline, this would not provide sufficient power to give reliable results. Moreover, because this will be a false-positive result for some subjects, further studies are needed to determine the impact of such a result on HRQoL.

### Implications

Following the baseline scan, this led to a clinically relevant increase in lung-cancer-specific distress in a substantial number of persons who underwent baseline screening, although the letter to participants clearly explained the meaning of an indeterminate test result (i.e. a very common small abnormality). Based on recent data from the NELSON trial and other lung cancer screening trials, the risk of lung cancer in this group is estimated to be <2.5% (Gohagan *et al*, 2004). Because distress levels may remain elevated until the result of the follow-up CT scan is known (e.g. 3 months in the NELSON), we recommend that the screening programme should be improved. For example, providing information about the small risk of having lung cancer in the letter might lead to a reduction in the lung-cancer-specific distress. Another approach could be to reduce the number of indeterminate test results by identifying certain subgroup of nodules with an increased cancer risk, or by a combination of imaging and proteomic or genomic biomarkers.

## Conclusions

This longitudinal study among participants of a lung cancer-screening programme showed in the short term that recipients of an indeterminate baseline screening result requiring a follow-up CT experience an increase in lung-cancer-specific distress, whereas the scores of recipients of a negative baseline screening result may be interpreted as a relief.

## Figures and Tables

**Table 1:** Characteristics of the respondents at T0

		**Baseline scan result**		
	**Total group (*n*=630)**	**Negative (*n*=489)**	**Indeterminate (*n*=141)**	***P* differences Negative/Indeterminate result**
Sex: male (%)	47.1	46.2	50.4	0.386^a^
Age in years: mean (s.d.), median	57.8 (5.5), 56.7	57.7 (5.5), 56.6	58.3 (5.6), 57.5	0.225^b^
Education				0.150^a^
1. Primary education (%)	9.3	8.9	10.8	
2. Lower vocational or lower secondary general education (%)	37.9	38.3	36.7	
3. Intermediate vocational or higher secondary general education (%)	25.1	23.4	30.9	
4. Higher vocational education or university (%)	27.7	29.4	21.6	
Marital status: Married/living with partner (%)	74.9	75.1	74.5	0.888^a^
				
*Smoking*				
Current smokers (%)	54.6	53.6	58.2	0.336^a^
Pack-years mean (s.d.), median	40.1 (17.8), 34.2	40.1 (18.2), 34.2	39.9 (16.3), 34.2	0.732^b^

Abbreviation: s.d.=standard deviation.

a *χ*^2^-test.

b Mann–Whitney *U*-test.

**Table 2:** Unadjusted mean (s.d.) HRQoL scores at the four assessment times, by baseline CT scan result (negative or indeterminate)

***N***	**T0**	**T1**	**T2**	**T3**
	**630**	**641**	**620**	**600**
	**Mean (s.d.)**	**Mean (s.d.)**	**Mean (s.d.)**	**Mean (s.d.)**
*SF-12 (PCS)*				
Total group	49.5 (8.7)			50.0 (8.2)
Negative	49.7 (8.4)			50.3 (8.3)
Indeterminate	48.5 (9.6)			48.9 (7.8)
				
*SF-12 (MCS)*				
Total group	51.9 (10.3)			51.6 (11.1)
Negative	51.9 (10.2)			51.6 (11.1)
Indeterminate	51.8 (10.6)			51.9 (11.0)
				
*EQ-5D, VAS*				
Total group	79.3 (13.7)	78.3 (12.9)	79.1 (12.3)	78.4 (13.7)
Negative	79.4 (13.8)	78.7 (12.6)	79.4 (12.2)	79.2 (13.4)
Indeterminate	79.1 (13.4)	76.8 (13.8)	78.3 (12.5)	75.0 (14.5)
				
*STAI-6*				
Total group	33.2 (8.6)	34.6 (8.6)	32.7 (8.8)	33.0 (9.2)
Negative	33.1 (8.4)	34.4 (8.5)	32.5 (8.8)	32.6 (9.2)
Indeterminate	33.6 (9.3)	35.2 (8.9)	33.5 (8.9)	34.8 (9.2)
				
*IES total score*				
Total group	4.2 (7.2)	5.9 (9.1)	4.5 (7.8)	3.6 (7.5)
Negative	4.1 (7.4)	5.8 (9.1)	4.5 (7.7)	2.4 (5.5)
Indeterminate	4.5 (6.5)	6.3 (9.1)	4.9 (8.4)	8.3 (11.3)
				
*IES intrusive*				
Total group	1.8 (3.4)	2.5 (4.0)	1.8 (3.6)	1.4 (3.3)
Negative	1.7 (3.5)	2.4 (4.0)	1.8 (3.5)	0.8 (2.4)
Indeterminate	2.0 (3.0)	2.7 (4.0)	2.0 (3.8)	3.5 (5.2)
				
*IES avoidance*				
Total group	2.4 (4.5)	3.5 (5.6)	2.7 (4.7)	2.2 (4.7)
Negative	2.4 (4.7)	3.4 (5.6)	2.7 (4.7)	1.5 (3.7)
Indeterminate	2.5 (4.1)	3.6 (5.7)	2.9 (4.9)	4.8 (6.9)

Abbreviations: T0=before trial randomisation, ie baseline HRQoL assessment; T1=1 week before the baseline CT scan; T2=1 day after the baseline CT scan; T3=2 months after the baseline CT scan; s.d.=standard deviation; SF-12=Short Form 12 (generic HRQoL); PCS=physical component summary; MCS=mental component summary; EQ-5D VAS=EuroQol questionnaire, visual analogue scale; STAI-6=Spielberger State-Trait Anxiety Inventory 6, IES=Impact of Event Scale.
